# Rural and urban exposures shape early life immune development in South African children with atopic dermatitis and nonallergic children

**DOI:** 10.1111/all.15832

**Published:** 2023-08-03

**Authors:** Nonhlanhla Lunjani, Anoop T. Ambikan, Carol Hlela, Michael Levin, Avumile Mankahla, Jeannette I. Heldstab‐Kast, Tadech Boonpiyathad, Ge Tan, Can Altunbulakli, Clive Gray, Kari C. Nadeau, Ujjwal Neogi, Cezmi A. Akdis, Liam O'Mahony

**Affiliations:** ^1^ Division of Dermatology University of Cape Town Cape Town South Africa; ^2^ APC Microbiome Ireland University College Cork Cork Ireland; ^3^ The Systems Virology Lab, Division of Clinical Microbiology, Department of Laboratory Medicine Karolinska Institute, ANA Futura Stockholm Sweden; ^4^ Division of Paediatric Allergy, Department of Paediatrics and Child Health University of Cape Town Cape Town South Africa; ^5^ The Division of Dermatology, Department of Medicine and Pharmacology Walter Sisulu University Mthatha Eastern Cape South Africa; ^6^ Swiss Institute of Allergy and Asthma Research (SIAF), University of Zurich Davos Switzerland; ^7^ Division of Immunology University of Cape Town Cape Town South Africa; ^8^ Department of Environmental Health Harvard T.H. Chan School of Public Health Boston MA USA; ^9^ Christine Kühne‐Center for Allergy Research and Education Davos Switzerland; ^10^ Department of Medicine University College Cork Cork Ireland; ^11^ School of Microbiology University College Cork Cork Ireland

**Keywords:** atopic dermatitis, children, environment, immune development, transcriptomics

## Abstract

**Background:**

Immunological traits and functions have been consistently associated with environmental exposures and are thought to shape allergic disease susceptibility and protection. In particular, specific exposures in early life may have more significant effects on the developing immune system, with potentially long‐term impacts.

**Methods:**

We performed RNA‐Seq on peripheral blood mononuclear cells (PBMCs) from 150 children with atopic dermatitis and healthy nonallergic children in rural and urban settings from the same ethnolinguistic AmaXhosa background in South Africa. We measured environmental exposures using questionnaires.

**Results:**

A distinct PBMC gene expression pattern was observed in those children with atopic dermatitis (132 differentially expressed genes [DEGs]). However, the predominant influences on the immune cell transcriptome were related to early life exposures including animals, time outdoors, and types of cooking and heating fuels. Sample clustering revealed two rural groups (Rural_1 and Rural_2) that separated from the urban group (3413 and 2647 DEGs, respectively). The most significantly regulated pathways in Rural_1 children were related to innate activation of the immune system (e.g., TLR and cytokine signaling), changes in lymphocyte polarization (e.g., TH17 cells), and immune cell metabolism (i.e., oxidative phosphorylation). The Rural_2 group displayed evidence for ongoing lymphocyte activation (e.g., T cell receptor signaling), with changes in immune cell survival and proliferation (e.g., mTOR signaling, insulin signaling).

**Conclusions:**

This study highlights the importance of the exposome on immune development in early life and identifies potentially protective (e.g., animal) exposures and potentially detrimental (e.g., pollutant) exposures that impact key immunological pathways.

## Graphical abstract

This study examines the molecular mechanisms and environment exposures underpinning the rural/urban immunological gradient in South African children with or without AD. Differences in animal exposures, home fuel use, time spent outdoors, and socioeconomic factors associated with differences in immune gene expression. One hundred and thirty‐two DEGs were observed in children with AD (including IGHE). UMAP clustering identified one urban and two rural groups characterized by differences in both lymphocyte and innate immune cell processes.Abbreviations: AD, atopic dermatitis; ALPL, alkaline phosphatase; CYP4F3, cytochrome P450 family 4 subfamily F member 3; DEFA3, defensin alpha 3; DEGs, differentially expressed genes; HSPA1A, heat shock protein family A (Hsp70) member 1A; HSPA1B, heat shock protein family A (Hsp70) member 1B; IGHE, immunoglobulin heavy chain constant epsilon; PGLYRP1, peptidoglycan recognition protein 1; UMAP, Uniform Manifold Approximation and Projection for Dimension Reduction

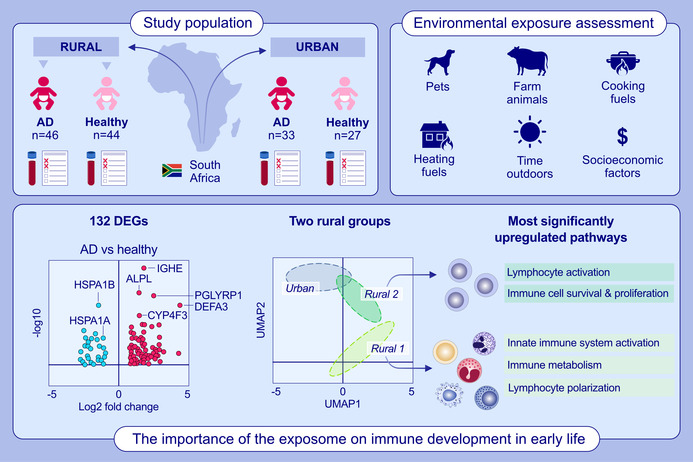

**Abbreviations**
AD: atopic dermatitis
AHR: aryl hydrocarbon receptor
AHRR: AHR repressor
GPCR: g protein‐coupled receptor
IL: interleukin
LILRs: inhibitory leukocyte immunoglobulin‐like receptors
MAPK: mitogen‐activated protein kinase
NF‐κB: nuclear factor kappa B
SPT: skin prick test
TARC: thymus and activation‐regulated chemokine
TLR: toll‐like receptor
TNF: tumor necrosis factor
VEGF: vascular endothelial growth factor

## 1 INTRODUCTION

There is an “immunological window of opportunity” early in life when the immune system is particularly responsive to environmental exposures (including infections, nutrition, and microbiome) that help establish the thresholds, patterns of reactivity, and functional trajectories that can have long‐term consequences including altered risk of immune‐mediated diseases.^1^, ^2^, ^3^, ^4^, ^5^ These interactions can be mediated partially via epigenetic mechanisms and ideally should promote appropriate immune responses that effectively defend against infection and tolerate the environmental otherwise harmless antigen/allergen exposures, with limited collateral damage to host tissue, and without any subsequent aberrant inflammatory or allergic reactions.^6^, ^7^, ^8^, ^9^

Multiple studies have shown that human immune traits are primarily influenced by environmental factors.^10^, ^11^ Some of the most important exposures in early life that determine functional programming of the infant immune system are associated with rural‐specific and urban‐specific factors. Rural or traditional farming lifestyles have been shown to modify innate (e.g., pattern recognition receptors—PRRs) and adaptive immune responses in children.^12^, ^13^, ^14^, ^15^ Differences in exposure to microbes, exposure to animals, dietary habits, use of cooking or heating methods that generate pollutants, and socioeconomic status have all been shown to influence the development of the early life immune system.^16^, ^17^, ^18^, ^19^, ^20^, ^21^, ^22^

We have previously shown that atopic dermatitis (AD) in South African children is associated with a distinct pattern of circulating cytokines (TARC, MCP‐4, and IL‐16) and elevated levels of specific IgE to food allergens and house dust mite.^4^ However, the most significant effects on circulating serum cytokine levels were due to rural and urban exposures in this South African cohort. In the current study, our aim was to further examine the molecular mechanisms and environment exposures underpinning the rural/urban immunological gradient in South African children with or without AD. We have, therefore, conducted high‐throughput mRNA sequencing (RNA‐Seq) on isolated peripheral blood mononuclear cells (PBMCs) from the children's cohort that has already been described. This includes rural children with AD (*n* = 46) or healthy (*n* = 44), and urban children with AD (*n* = 33) or healthy (*n* = 27).

## 2 METHODS

### 2.1 Study design

This study was originally designed as a matched case–control study to evaluate the differences in children with or without AD in rural and urban settings. All participants were from the ethnolinguistic AmaXhosa population. The AmaXhosa nations live in the Cape region of South Africa. The urban AmaXhosa study participants were recruited from the Cape Town metropole and predominantly reside in urban townships, while the rural AmaXhosa study participants were recruited from the Umtata district and predominantly reside in rural reserves and live in homesteads (cluster of houses with extended family) practicing subsistence crop and pastoral farming. All children with AD had a dermatologist diagnosis of AD. The UK working party criteria was used for study inclusion with 100% of children being positive for itch, a history of flexural AD, and a history of dry skin and visible flexural dermatitis. The objective SCORAD index was used to grade disease severity and participants with moderate to severe disease were included in the study. The median SCORAD for rural AD was 43.0 and the median SCORAD for urban AD was 43.5. All participants with AD were on soap substitutes, emollients, and topical steroid therapy only. None were on systemic treatments or biologicals. Urban children with moderate to severe atopic dermatitis (*n* = 33) were enrolled at the Paediatric Dermatology Clinic, Red Cross Children's Hospital, Cape Town. Healthy nonallergic, nonfood sensitized (on SPT screening) urban participants (*n* = 27) were enrolled from randomly selected crèches in the Cape Town metropole. Similarly, children from a rural setting with AD (*n* = 46) were enrolled at the dermatology clinic at the Nelson Mandela Academic Hospital, Umthatha. Their nonallergic healthy control counterparts (*n* = 44) were randomly selected at study sites based at village clinics in the Mqanduli district of Umthatha. They were 15–35 months old with balanced age and gender between the groups and normal weight within their age standards (Table S1). All participants were seen at one time point for questionnaire, SPT, clinical assessment, and blood sample collection. Detailed standardized questionnaires were completed with the parent/guardian to obtain data on family history of atopy, household size, family income, infant exposures, medication use, and environmental exposures, as previously described.^23^ This clinical study received Human Research Ethics Committee approval (HREC 451/2014) and was conducted in accordance with the declaration of Helsinki and written informed consent was obtained from parents/guardians of all participants prior to inclusion in the study.

### 2.2 Cell isolation and cryopreservation

Whole blood samples in lithium heparin tubes were collected from participants (*n* = 150) and PBMCs were isolated according to standard protocols. Briefly, the separation of PBMCs was achieved using Ficoll‐Paque 1.077 g/mL density gradient centrifugation. Washed PBMCs were resuspended in freezing medium (DMSO, heat‐inactivated FCS, and RPMI). Cells were then transferred to a −80 C freezer and thereafter liquid nitrogen the following day.

### 2.3 RNA isolation & sequencing

Total mRNA was isolated from PBMCs using the RNeasy Mini Kit (Qiagen), and RNA levels were quantified by NanoDrop (Thermo Fisher Scientific). RNA integrity was confirmed using the Agilent 2100 BioAnalyzer (Agilent). Libraries were prepared with Illumina's Truseq stranded mRNA library prep kit with polyA enrichment. RNA‐Seq analysis was performed on samples from all children (*n* = 150) using HiSeq 4000.

### 2.4 RNA‐Seq data preprocessing

All the raw fastq files generated were processed using the RNA‐Seq pipeline from the NF‐core framework.^24^ The pipeline uses Trim Galore for adapter trimming with default options.^25^ Human reference genome version GRCh38 obtained from Ensembl was used for alignment and read count estimation was performed using aligner option star_salmon provided by the pipeline. Gene level estimated count and TPM (transcript per million) normalized count of genes belonging to biotypes namely, protein‐coding, lincRNAs, IG gene, and TR gene were used for all the downstream analysis. Details of the biotypes can be viewed at https://www.ensembl.org/info/genome/genebuild/biotypes.html. Dimensionality reduction of the data was performed using Uniform Manifold Approximation and Projection for Dimension Reduction (UMAP) method implemented in the R package umap v0.2.7.0 for visualizing the sample distribution. TPM normalized gene expression matrix was used for UMAP dimensionality reduction.

### 2.5 Digital cell quantification (DCQ)

Digital cell quantification (DCQ) estimates proportion of various cell types present in the samples based on bulk RNA‐Seq data. As the data was generated from PBMCs, differences in cell type abundance will significantly influence and bias the gene expression profile of the samples. To adjust for this bias, we have used digital cell quantification (DCQ) results while performing the differential expression analysis to reduce the changes in abundance of specific immune cell types in each sample. The DCQ procedure uses cell type specific gene expression data of 18 different cell types downloaded from Human Protein Atlas and signature genes of each cell type collected from CellMarker and PangloDb.^26^, ^27^ A deconvolution algorithm was then applied, which is adapted from Estimating the Proportions of Immune and Cancer cells (EPIC) method.^28^

### 2.6 Sample clustering

K‐means algorithm implemented in R was used to identify the sample clusters. R function K‐means was used for the analysis. The top 5000 highly varying genes based on median absolute deviation were selected for generating the sample clusters. Euclidean distance between each samples were computed using get_dist function from the R package factoextra v1.0.7. Number of possible clusters were decided based on average silhouette width calculated using the function fviz_nbclust from the R package factoextra v1.0.7.

### 2.7 Differential gene expression and gene‐set enrichment analysis

Differential gene expression analysis (DGE) identified genes upregulated or downregulated using Bioconductor package DESeq2 v1.34.0.^29^ Confounding factors including cell type proportions, age, and gender were adjusted while performing differential expression analysis. Bioconductor package RUVSeq v1.28.0 was used to compute the factor of unwanted variation and added to the DESeq2 design matrix.^30^ A gene was considered to be significantly regulated when adjusted *p* < .05 and log2 fold change ≥1 (two times the expression difference).

Gene‐set enrichment analysis was performed using R package piano v2.10.0 to identify pathways of interest.^31^ KEGG pathway gene‐sets and gene‐level statistics, and log2 fold change values were used to identify regulated pathways in each of the pairwise comparisons. KEGG pathways belong to the categories Metabolism, Environmental Information Processing and Organismal Systems were considered in the analysis.

## 3 RESULTS

### 3.1 PBMC gene expression profile associated with AD

**FIGURE 1:**
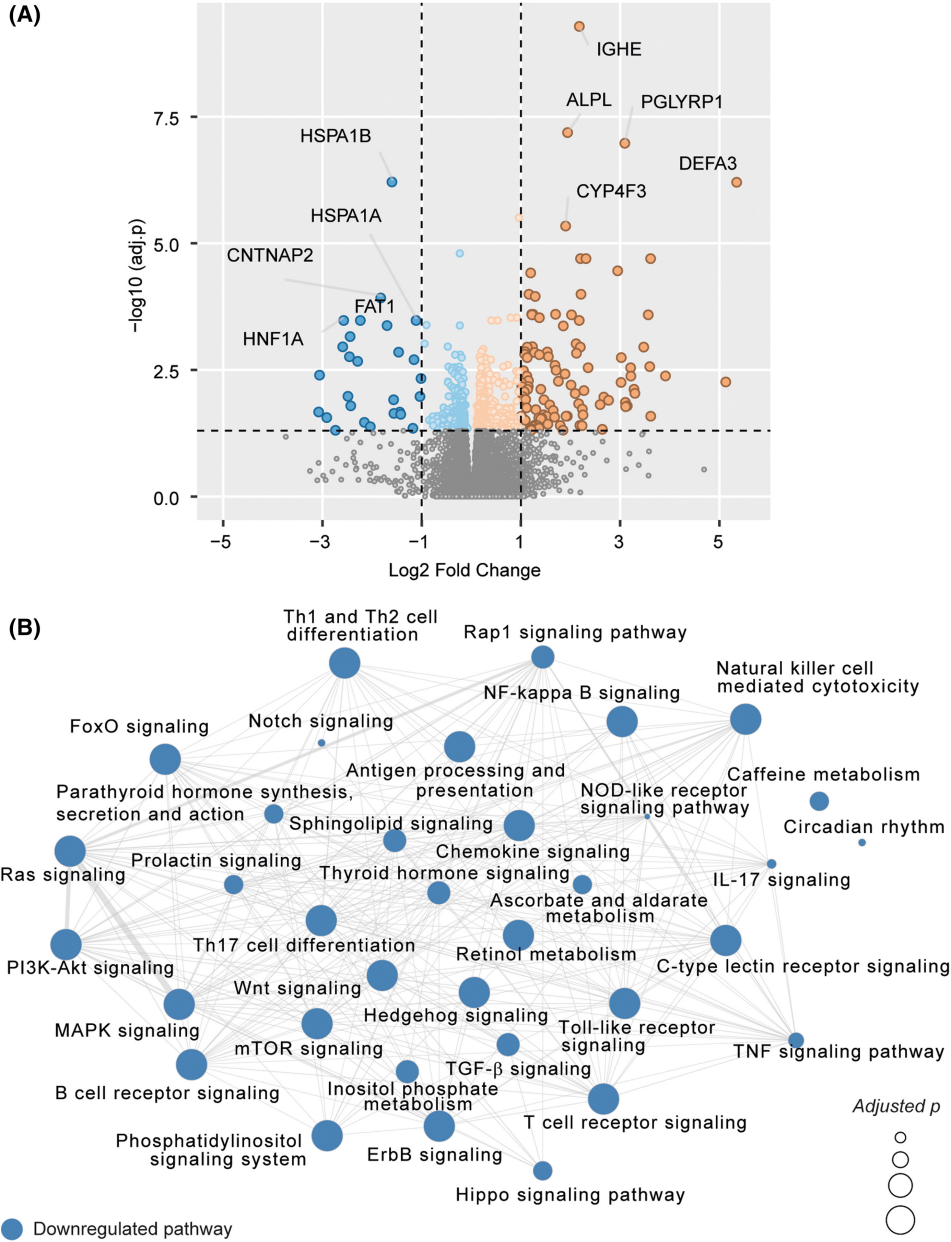
(A) Volcano plot showing differential gene expression analysis results in Healthy versus Atopic Dermatitis (AD). Positive Log2 Fold Change values represent upregulation and negative values represent down‐regulation in AD compared to Healthy. (B) Pathway enrichment analysis results. The network shows significantly downregulated pathways (adjusted *p* < .05) identified in AD. No significantly upregulated pathways were found. Node size is relative to negative log10 scaled adjusted *p*‐values. Edges represent overlap of at least 10 genes between the pathways.

A total of 132 (adjusted *p* < .05 and |log2 Fold change| ≥ 1) differentially expressed genes (DEGs) were identified in PBMCs from children with AD (*n* = 79) versus without AD (*n* = 71; Figure 1A). While relatively few genes were altered in PBMCs of AD patients, the most highly upregulated gene was *IGHE*, encoding the heavy chain constant region of IgE (adjusted *p* = 5.2 × 10^−10^), reinforcing the link between AD and atopic mechanisms. Gene expression of AD‐associated mediators such as IL‐21, IL‐21 receptor, IL‐5, and IL‐9 receptor were altered in children with AD using unadjusted *p*‐values, but adjusted *p*‐values were no longer significant.^32^, ^33^, ^34^ 62% of the AD patients had a skin prick test (SPT) positive reaction to at least one of the food allergens tested (egg, milk, soy, wheat, fish [cod], peanut, or hazelnut). However, there were no differences in the RNA‐Seq data in children with AD and a positive SPT reaction, versus children with AD and a negative SPT reaction (Figure S1). Pathway enrichment analysis identified a range of lymphocyte patterns (e.g., TH1, TH2, and TH17 differentiation) and innate signaling patterns (e.g., TLR signaling) that were downregulated in AD compared to healthy volunteers (Figure 1B). These AD gene expression profiles were observed in both rural and urban children.

### 3.2 Living environment dominates PBMC gene expression clustering

**FIGURE 2:**
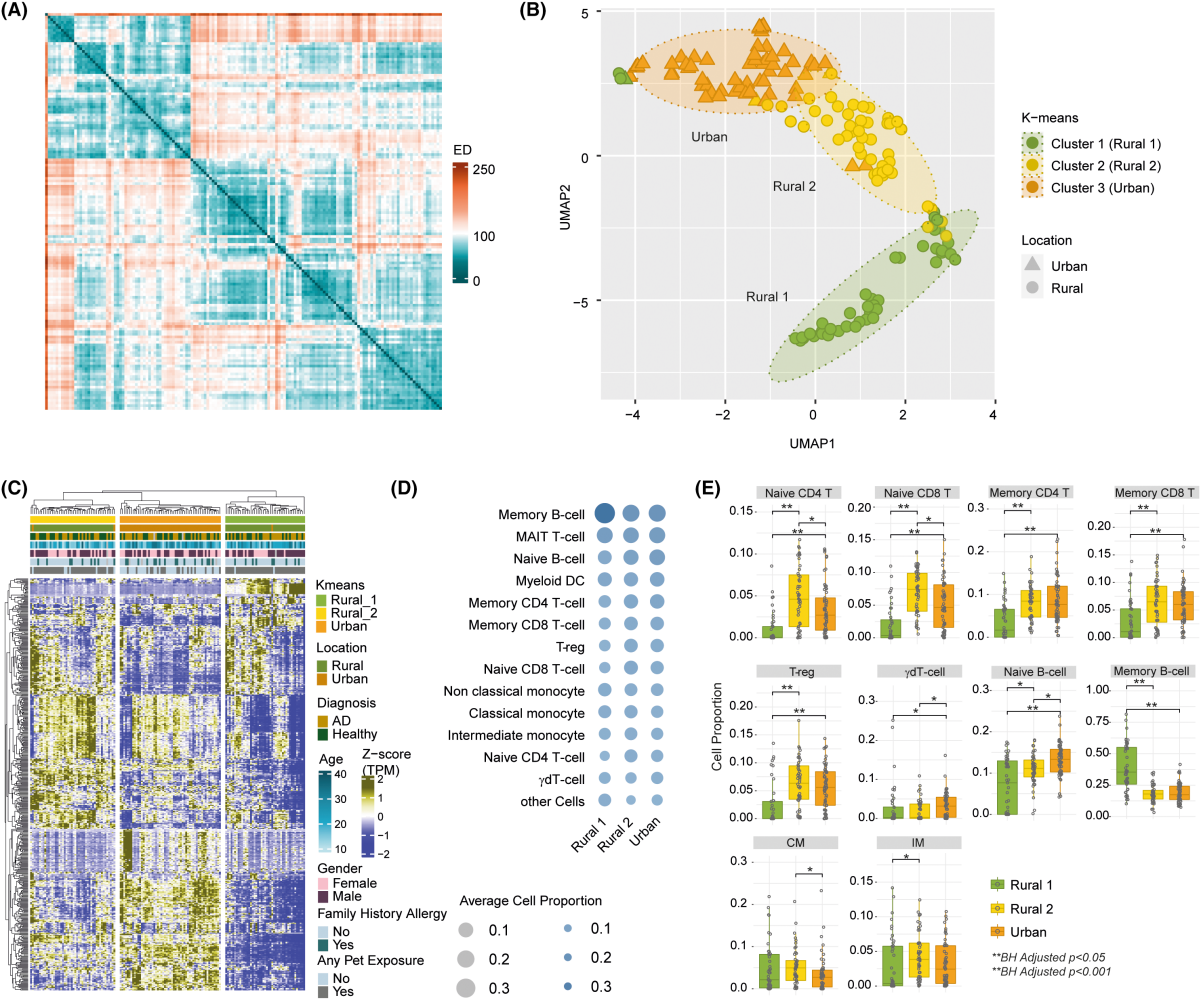
(A) Heatmap of sample—sample similarity based on Euclidean Distance (ED) metric created with top 5000 highly varying genes. Column and row are samples. Lower ED represents higher similarity between samples. The heatmap shows subpopulations in the cohort. (B) Three subpopulations identified using K‐means algorithm and top 5000 highly varying genes as input. The majority of Urban samples are in one cluster and Rural samples formed two separate clusters, named Rural 1 and Rural 2. (C) Expression pattern of top 500 highly varying genes among all the samples showing differences between the subpopulations identified. Column annotation shows the subpopulations and other clinical characteristics corresponding to each sample. (D) Digital cell quantification results. Bubble size and color gradient are relative to average cell proportions computed for samples in each subpopulation. (E) Box plot of cell types showed significant difference (adjusted *p* < .05) between subpopulations identified by Mann–Whitney *U* test.

Data‐driven sample clustering identified groups of children with similar transcriptomic landscapes. Visualization of similarities using Euclidean distance metric showed evidence for distinct clusters of children and average silhouette width identified three clusters in the total cohort (Figure 2A). K‐means‐based clustering found that all children (except three) from the urban group formed one cluster, while there were two subclusters of children in the rural setting, termed Rural_1, and Rural_2 (Figure 2B). The heatmap of the top 500 highly varying genes showed unique expression patterns for each of the three K‐means clusters identified (Figure 2C). There were no differences in age or gender between the groups. Importantly, the clustering was primarily driven by the location of the child, and not the presence of AD, demonstrating the strong environmental and living conditions effect on the immune system of the growing child.

Analysis of the estimated proportions of various cell types in the samples by digital cell quantification (DCQ) revealed highly significant differences in cell type abundances between rural and urban children (Figure 2D). Children in the Rural_1 group had the highest levels of memory B cells, but the lowest levels of naïve and memory CD4^+^ and CD8^+^ lymphocytes, T regulatory cells and naïve B cells. In contrast, Rural_2 children had the highest levels of naïve CD4^+^ and CD8^+^ lymphocytes, and classical and intermediate monocytes. Urban children had the highest levels of γδ T cells (Figure 2E). There were no differences in the levels of NK cells, plasmacytoid DCs, MAIT cells, myeloid DCs, and nonclassical monocytes. There was no statistically significant difference in cell type specific gene signatures in children with AD compared to their healthy counterparts.

### 3.3 Environmental factors associated with PBMC gene expression clustering

Several exposures were significantly related to the rural and urban gene expression clusters. The most statistically significant differences were related to animal exposures and sunlight exposure (reflecting time spent outdoors) during both winter and summer in the rural group (Table 1). In addition, there were differences in family income between the rural and urban groups. While there were no significant differences in the questionnaire data that could explain the two different rural clusters, only Rural 1 children were significantly different from the urban cluster for birth mode (highest rate of vaginal delivery for Rural 1). Only Rural 2 infants were significantly different from the urban cluster for lower rates of peanut and amasi (a fermented drink) consumption and differences in the type of heating fuel used (Table 1).

**TABLE 1:** Exposures that significantly associate with the UMAP clustering are illustrated. The adjusted *p*‐values are shown for those comparisons that are significant, and for those comparisons that show *p*‐value trends between different groups. An adjusted *p*‐value greater than 0.20 (adjusted *p* > .20) is illustrated as nonsignificant (NS).

	Rural versus Urban	Rural 1 versus Urban	Rural 2 versus Urban	Rural 1 versus Rural 2
Cat exposure	**0.03**	**0.007**	0.14	NS
	Rural highest	Rural 1 highest		
Dog exposure	**3** × **10** ^**−4**^	**8** × **10** ^**−4**^	**2** × **10** ^**−3**^	NS
	Rural highest	Rural 1 highest	Rural 2 highest	
Farm animals—child	**2** × **10** ^**−15**^	**6** × **10** ^**−13**^	**2** × **10** ^**−12**^	NS
	Rural highest	Rural 1 highest	Rural 2 highest	
Farm animals—mother	**8** × **10** ^**−16**^	**3** × **10** ^**−13**^	**3** × **10** ^**−12**^	NS
	Rural highest	Rural 1 highest	Rural 2 highest	
Peanut consumption	**0.002**	0.06	**0.002**	NS
	Urban highest		Urban highest	
Amasi consumption	**0.002**	NS	**0.01**	NS
	Urban highest		Urban highest	
Winter sun	**6** × **10** ^**−13**^	**1** × **10** ^**−11**^	**9** × **10** ^**−9**^	NS
	Rural highest	Rural 1 highest	Rural 2 highest	
Summer sun	**2** × **10** ^**−10**^	**1** × **10** ^**−11**^	**2** × **10** ^**−4**^	NS
	Rural highest	Rural 1 highest	Rural 2 highest	
Income	**6** × **10** ^**−6**^	**2** × **10** ^**−4**^	**3** × **10** ^**−5**^	NS
	Urban highest	Urban highest	Urban highest	
Antibiotics	**0.05**	0.10	NS	NS
Frequency	Rural highest			
Paracetamol frequency	**0.05**	NS	0.07	NS
	Urban highest			
Delivery mode (vaginal delivery)	**0.03**	**0.04**	0.16	NS
	Rural highest	Rural 1 highest		
Cooking fuel	**1** × **10** ^**−7**^	**2** × **10** ^**−8**^	**1** × **10** ^**−4**^	NS
Paraffin	Rural highest	Rural 1 highest	Rural 2 highest	
Open fire	Rural highest	Rural 1 highest	Rural 2 highest	
Electricity/gas	Urban highest	Urban highest	Urban highest	
Heating fuel	**0.03**	NS	**0.005**	NS
Paraffin/kerosene	Rural highest		Rural 2 highest	
Wood	Rural highest		Rural 2 highest	
Electricity/gas	Urban highest		Urban highest	

Bold values indicate statistical significance (*p* < 0.05)

### 3.4 Differential gene expression analysis

**FIGURE 3:**
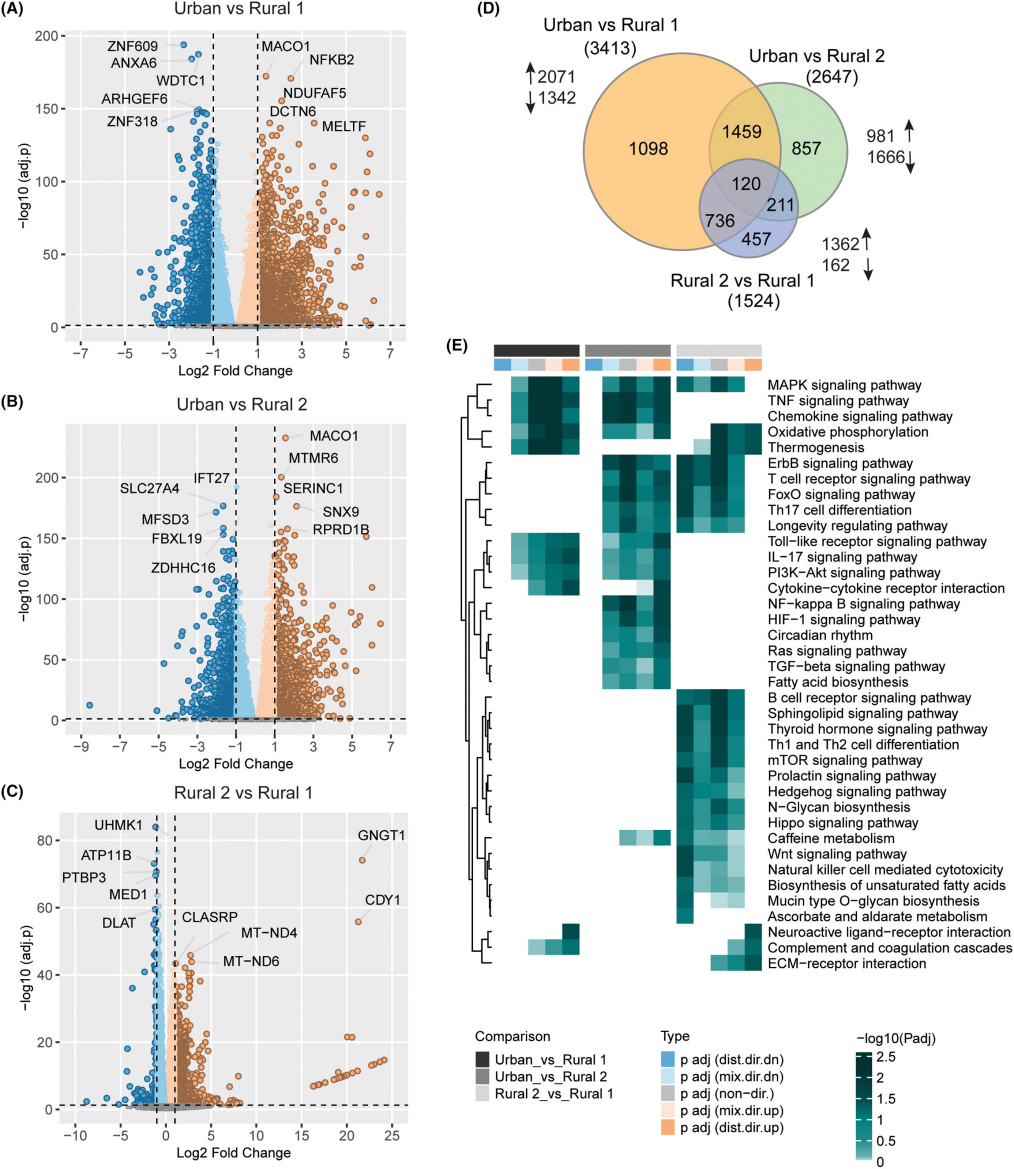
(A) Volcano plot showing differential gene expression analysis results in Urban versus Rural 1. Positive Log2 Fold Change values represent upregulation and negative values represent down‐regulation in Rural 1 compared to Urban. Top 5 upregulated and downregulated genes (adjusted *p* < .05, Log2 Fold Change >1) are labeled. (B) Volcano plot showing differential gene expression analysis results in Urban versus Rural 2. Positive Log2 Fold Change values represent upregulation and negative values represent down‐regulation in Rural 2 compared to Urban. Top 5 upregulated and downregulated genes (adjusted *p* < .05, Log2 Fold Change >1) are labeled. (C) Volcano plot showing differential gene expression analysis results in Rural 2 versus Rural 1. Positive Log2 Fold Change values represent upregulation and negative values represent downregulation in Rural 2 compared to Rural 1. Top 5 upregulated and downregulated genes (adjusted *p* < .05, Log2 Fold Change >1) are labeled. (D) Venn diagram of significantly expressed genes (adjusted *p* < .05, Log2 Fold Change >1) in each pairwise comparison. (E) Pathway enrichment analysis results. The heatmap shows significantly upregulated and downregulated pathways (adjusted *p* < .1) in each comparison. The heatmap visualizes negative log scaled adjusted *p*‐values of different directionality classes computed by the enrichment analysis. Nondirectional *p*‐values are generated based on gene‐level statistics alone without considering the expression direction. The mixed‐directional *p*‐values are calculated using subset of gene‐level statistics of up‐ and downregulated genes, respectively, for mixed‐directional up and down. Distinct directional up and distinct directional down *p*‐values are calculated from gene statistics with expression direction. Distinct class is used to define upregulated and downregulated pathways. The column annotation includes directionality of pathways and corresponding differential expression analysis.

Following adjustments for confounding factors, genes that were significantly upregulated or downregulated between the three clusters of children were identified (Figure 3A–C). The primary driver of gene expression changes is the living environment of the child as clearly seen by the volcano plots and Venn diagram. The largest number of DEGs was observed for the Rural_1 to Urban comparison (*n* = 3413), with slightly fewer DEGs for the Rural_2 to Urban children comparison (*n* = 2647), while relatively low number of DEGs was observed for the Rural_1 to Rural_2 comparison (*n* = 1524; Figure 3D). Distinct immune pathway enrichments were observed for each of these comparisons (Figure 3E).

**FIGURE 4:**
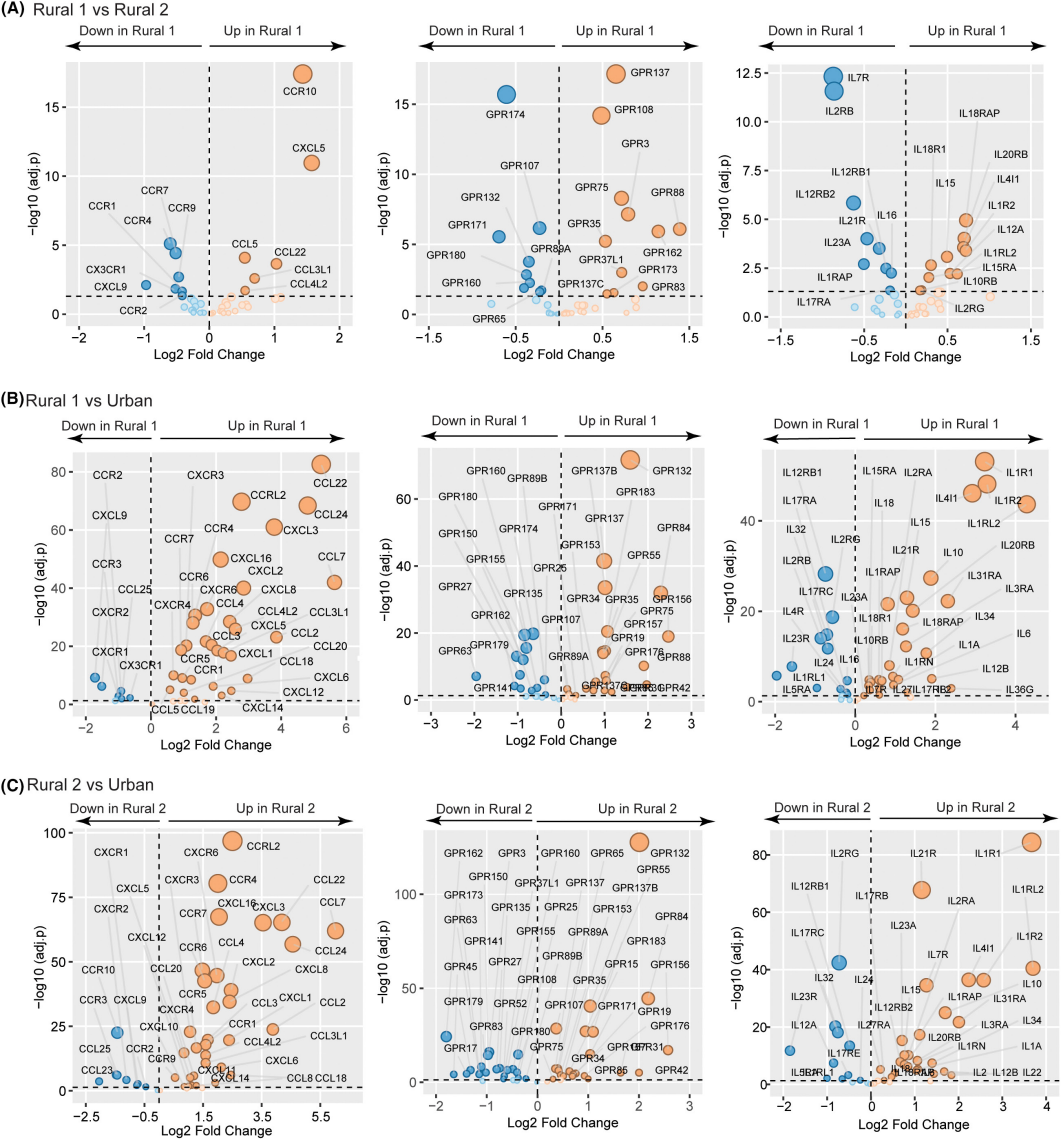
Significant differences (adjusted *p* < .05) in PBMC chemokine and chemokine receptors, GPCRs, interleukins, and interleukin receptors are illustrated when comparing children in the Rural_1 versus Rural_2 clusters (A), Rural_1 versus Urban clusters (B), and Rural_2 versus Urban clusters (C). Adjusted *p*‐value (*y*‐axis) and fold change are plotted for each significant gene.

We also specifically examined the expression levels of chemokines and their receptors, interleukins, and their receptors, and G protein‐coupled receptors (GPCRs; Figure 4). When comparing the two rural subgroups, several immunologically relevant genes were differentially expressed (Figure 4A). Children in the Rural_1 group had the highest expression levels of CCR10, GPR137, and IL‐15, while children in the Rural_2 group had increased expression levels of CCR7, GPR174, and IL‐23A. However, a much larger number of genes were differentially expressed when comparing the Rural_1 or Rural_2 groups with the Urban group (Figure 4B,C).

Previously, we showed that serum levels of many chemokines were significantly elevated in rural compared to urban children.^4^ Similarly, we noted significant upregulation of multiple chemokines and chemokine receptors in PBMCs from both rural children groups compared to urban children (Figure 4B,C). A substantially smaller number of chemokine and chemokine receptor genes were upregulated in PBMCs from urban children.

GPCRs have been shown to exert significant modulatory effects on immune cell activity and polarization. Multiple GPCRs were differentially regulated between rural and urban children with similar numbers of GPCR genes either being upregulated in PBMCs from rural children or PBMCs from urban children. The most significantly upregulated GPCR gene in rural children was GPR132, which has been shown to be involved in the control of autoimmune responses and can be activated by microbiota‐derived metabolites.^35^, ^36^

In general, many more interleukin‐related genes were upregulated in the two rural groups compared to the urban children. For example, rural children displayed significantly elevated levels of IL‐1 receptor subtypes (IL‐1R1, IL‐1R2, IL‐1RL2, IL‐1RAP, and IL‐1RN) suggesting higher levels of IL‐1 signaling in rural children. Importantly, IL‐10 gene expression was significantly upregulated in rural children compared to urban children. In contrast, urban children had the highest expression levels of IL‐2 receptor subunits (IL‐2Rγ and IL‐2Rβ) and genes related to TH17 signaling (IL‐23R, IL‐17RC, IL‐17RE, and IL‐17RA).

Other immunologically relevant genes that were differentially expressed include the inhibitory leukocyte immunoglobulin‐like receptors (LILRs). Leukocyte immunoglobulin‐like receptor B1 (LILRB1), LILRB2, LILRB3, and LILRB4 were significantly upregulated (adjusted *p* < .05) in PBMCs from both rural subgroups compared to the urban children group. In addition, expression levels of genes required for histamine synthesis, histamine degradation, and histamine receptors were significantly different between rural and urban children. Histidine decarboxylase (HDC) and histamine receptor 2 (H_2_R) were significantly elevated in PBMCs from urban children (adjusted *p* < .05), while H_1_R and histamine N‐methyltransferase (HNMT) were significantly elevated (adjusted *p* < .05) in PBMCs from rural children.

Finally, aryl hydrocarbon receptor (AHR) and AHR repressor (AHRR) gene expression were significantly increased in the Rural_2 group compared to Urban children, while AHRR (but not AHR) was significantly increased in the Rural_1 group compared to Urban children (adjusted *p* < .05, Figure S2). AHRR gene expression positively correlated with summer and winter sunlight exposure (adjusted *p* < .05) and was significantly higher expressed for children living in homes using fires or paraffin for cooking, or paraffin/wood/coal for heating compared to children living in homes using electricity or gas for cooking and heating (adjusted *p* < .05). No significant correlations were found with AHR gene expression.

### 3.5 Co‐expression network analysis

While many individual genes were significantly different between the groups, a more complete understanding of the biological changes can be inferred from weighted co‐expression network analysis. Gene co‐expression communities that capture the transcriptomics signatures specific to each cohort were identified and the corresponding functions were investigated.

**FIGURE 5:**
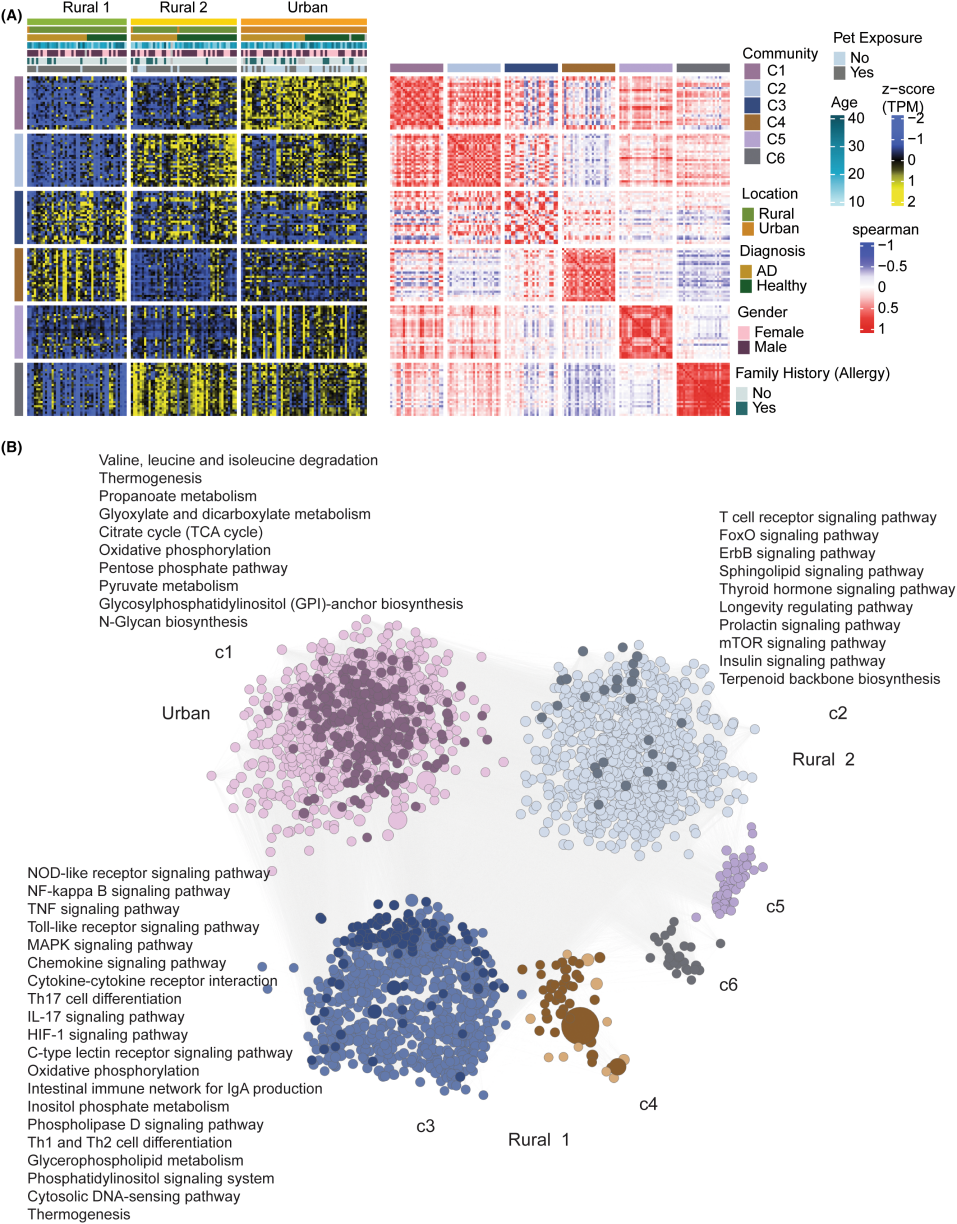
(A) Global weighted co‐expression network topology analysis. The heatmap shows expression of top 25 nodes from each of the six co‐expression gene communities identified. The nodes were ranked based on betweenness centrality. Centrality measurement defines the importance of each node in the existence of the network. Column annotation shows the cohort and other clinical parameters corresponding to each sample. Row annotation denotes the gene communities. The communities were further examined for their association with the three cohort subpopulations. Ratio of presence of significantly upregulated genes in a subpopulation compared to other two subpopulations (signature genes) was used to define the corresponding subpopulation‐specific community. The analysis found that gene co‐expression community 1 (C1) and community 5 (C5) were specific to Urban children, gene co‐expression community 2 (C2) was specific to Rural_2 subpopulation and gene co‐expression community 3 and 4 (C4 and C4) were specific to Rural_1 population. The second heatmap shows the association between each of the communities calculated by Spearman correlations. (B) Network visualization of the top 25% nodes of six co‐expression communities. Betweenness centrality was used to rank the nodes. Nodes represent genes belonging to communities and edges represent significant (adjusted *p* < 1e‐5) positive Spearman's correlations among the genes. Node size is relative to betweenness centrality of the node. Dark‐colored nodes denote the subpopulation‐specific signature genes. Significant pathways (adjusted *p* < .1) associated with each community are labeled.

This analysis identified six communities (c1–c6) of highly intercorrelated gene expression (Figure 5A). Gene expression patterns that characterize urban children were found within communities c1 and c5, communities c3 and c4 were associated with the Rural_1 group, while community c2 was enriched in the Rural_2 children. The gene co‐expression community specific to the urban children (c1) shows evidence of metabolic compromise, as these pathways suggest a unique pattern of PBMC metabolism (Figure 5B). The pathways that were most significantly regulated in Rural_1 children were related to innate activation of the immune system (e.g., TLR signaling, NF‐κB signaling, MAPK signaling), cytokine (including TNF) and chemokine signaling, changes in lymphocyte polarization (e.g., TH17 cells), and immune cell metabolism (i.e., oxidative phosphorylation). The Rural_2 gene co‐expression c2 community suggests ongoing lymphocyte activation (e.g., T cell receptor signaling), with changes in immune cell survival and proliferation (e.g., mTOR signaling, insulin signaling).

## 4 DISCUSSION

RNA‐Seq analysis of PBMCs from this South African children's cohort revealed highly significant changes in immune cell gene expression profiles that were heavily dependent on the rural versus urban environment of the child. AD was also associated with distinct changes in circulating immune cell gene expression, but far less than the changes induced by environmental exposures, perhaps due to many, but not all, of the inflammatory effector responses being restricted to the skin or the lack of gene expression for barrier factors by PBMCs.^37^, ^38^

There is a growing appreciation of ethnogenetic background in shaping immune activation in AD and disease outcomes.^39^, ^40^ In addition, region‐specific diversity in environmental exposures (e.g., *S. aureus* carriage), socioeconomic factors, diet, and lifestyles can expose gene–environment interactions that yield very different outcomes when taking ethnicity into consideration. Upregulation of IgE expression in this cohort highlights the importance of T_H_2 responses in AD that are shared between African, European, and Asian studies.^41^ We also noted reduced expression of T_H_1‐, T_H_2‐, and T_H_17‐associated genes in the peripheral circulation likely reflecting mass migration of these cells into the skin compartment, which corresponds with the previously described T_H_1‐, T_H_2‐, and T_H_17‐high skin biopsy profile in early onset AD.^42^ As no differences in pathway enrichment for the T_H_22 axis were observed (increased skin T_H_22 responses were previously only detected in adult‐onset, but not infant AD), the AD profile described in our cohort aligns with age‐specific features of the disease that have been documented in pediatric AD elsewhere. However, the relative contribution of T_H_1, T_H_17, and T_H_22 responses to AD across ethnogenetic backgrounds certainly requires further study, especially in well‐matched multiethnic cohorts with adequate power to detect differences.

Unsupervised clustering of children's PBMC gene expression profiles revealed two rural subgroups that were separated from children within the urban setting. These two rural gene expression patterns may represent the transition from a truly rural gene expression signature (rural group 1) to an intermediate gene expression signature (rural group 2), to the urban pattern of immune cell gene expression. The average cell proportions identified using digital cell quantification were more similar for rural group 2 and the urban children, supporting the immune transition hypothesis. Currently, we do not know if these environmental effects on immune cell gene expression are related only to specific exposures characteristic of this cohort, and further studies in geographically unrelated cohorts are required in order to determine the generalizability of our findings. A recent Swedish study did not find any significant differences in the proportions of major immune lineages between anthroposophic (families with higher levels of home delivery, lower frequency of body washing, prolonged breastfeeding, organic diet with live lactobacilli, and restricted use of antibiotics, antipyretics, and vaccinations) non‐IgE sensitized and nonanthroposophic IgE sensitized children, suggesting that the rural–urban differences in exposures that influence immune cell populations in this South African cohort may be missing in the North European setting.^1^ Animal exposures, sunlight exposures and type of cooking fuel were significantly different between rural and urban South African children but were not significantly different between the two rural subgroups. Delivery mode was significantly different only for rural group 1 (not for rural group 2) compared to urban children and c‐section delivery can significantly influence the development of the early life microbiota, thereby impacting immune development.^43^, ^44^ The use of wood or coal as home heating fuel was only significantly different for rural group 2 compared to urban children, which may also have effects on immune cell gene expression. However, it is likely that other factors not recorded in this study (e.g., outdoor pollution level, detailed dietary habits, etc.) may also drive the differences in gene expression between the two rural subgroups.

Multiple pathways that are well described to regulate or suppress aberrant inflammatory immune responses were more highly expressed in rural children. IL‐10 gene expression was highly upregulated in rural children's PBMCs, and IL‐10 potently limits effector functions of antigen‐presenting cells and lymphocytes.^45^ Gene expression of additional IL‐10 family members (IL‐20R and IL‐22) similarly elevated. Inhibitory leukocyte immunoglobulin‐like receptors were increased in rural children (LILRB1 to LILRB4). These receptors are expressed on immune cells where they bind to MHC class I molecules on antigen‐presenting cells and transduce a negative signal that inhibits stimulation of an immune response, thereby modulating cell activation thresholds and maintaining immune tolerance.^46^, ^47^, ^48^ G protein‐coupled receptors (GPCRs) are involved in a wide array of physiological functions including important roles in regulating immune responses, attenuating inflammation, and promoting return to homeostasis.^49^ A surprisingly large number of GPCRs were differentially expressed in PBMCs from rural and urban children. The GPCRs with immune regulatory functions that were elevated in PBMCs from rural children include GPR132 (inhibits autoimmune responses), GPR183 (protective role in SLE and important for germinal center reactions), GPR55 (negative regulator of γδ T cell migration), GPR17 (negative regulator of inflammatory cell recruitment and modulates TH2/TH17 cytokine expression), GPR84 (regulates TH2 effector cell function), GPR35 and GPR135 (activated by tryptophan metabolites), GPR31 (essential in the induction of oral tolerance by maintaining IL‐10 producing intestinal RORγt^+^ Foxp3^+^ Treg cells), GPR171 (suppressor effects on T‐cell‐mediated effector responses), GPR15 (regulates preferential homing of Foxp3^+^ Treg cells to the large intestine), and GPR65 (regulates immune cell migration and maintains epithelial barrier homeostasis).^50^, ^51^, ^52^, ^53^, ^54^, ^55^, ^56^, ^57^, ^58^, ^59^, ^60^ The majority of GPCRs that are upregulated in PBMCs from urban children have not yet been functionally assessed. However, exceptions include GPR3 (expressed by activated effector and regulatory T cells), GPR174 (restrains Foxp3^+^ Treg cell development, activation, and immune regulatory activity), and GPR108 (negative regulator of TLR responses but potent activator of NF‐κB when overexpressed).^61^, ^62^, ^63^, ^64^ Overall, the combination of regulatory cytokines, inhibitory receptors, and GPCRs suggest a higher level of immunoregulatory pathway activation in rural compared to urban children.

AHR is well known for sensing xenobiotic agents and xenobiotic metabolism in the liver.^65^, ^66^ However, it is also expressed within epithelial barrier tissues and associated immune cells, where AHR signaling (often in response to nonxenobiotic compounds such as microbiota‐derived indoles) contributes to maintenance of regulatory cell function and TH17 differentiation.^36^ AHRR is expressed in response to AHR activation and subsequently functions to counter‐regulate AHR target genes.^67^ It also plays a major role in immune cell differentiation and function.^68^ Interestingly, AHRR expression profiles did not mirror AHR expression in this cohort, suggesting that AHR may be consistently expressed, and inducible AHRR gene expression may better reflect activation of the AHR. In addition, AHRR may be selectively expressed by specific immune cell subsets or may be upregulated via AHR‐independent mechanisms.^68^ Regardless, increased expression of AHRR by rural children suggests increased exposure to AHR activating agents such as pollutants outside the home, or inside the home, or maybe in response to a different diet and microbiota metabolism.

In conclusion, the predominant influences on the peripheral blood immune cell transcriptome in children are related to early life environmental exposures and lifestyle factors. Protective (e.g., microbiota and animal) exposures and potentially detrimental (e.g., virus infection and pollutants) exposures shape the early life innate and adaptive immune response. Understanding these mechanisms will progress our appreciation for the importance of environment on immune development and prevention of chronic immune‐mediated disorders. These discoveries will also underpin the development of novel diagnostic markers and translational targets for more specific and safe modulation of immune activity, both within the skin, systemically and within other organs.

## AUTHOR CONTRIBUTIONS

Nonhlanhla Lunjani, Jeannette I. Heldstab‐Kast, Can Altunbulakli, Tadech Boonpiyathad, Anoop T. Ambikan, Ujjwal Neogi, and Liam O'Mahony performed laboratory assays and contributed to data analysis. Nonhlanhla Lunjani, Clive Gray, Carol Hlela, Michael Levin, and Avumile recruited subjects and obtained samples. Nonhlanhla Lunjani, Clive Gray, Carol Hlela, Michael Levin, Kari C. Nadeau, Ujjwal Neogi, Cezmi A. Akdis, and Liam O'Mahony obtained funding, supervised the work, and wrote the manuscript. All authors have read and approved the manuscript.

## CONFLICT OF INTEREST STATEMENT

Dr.Nadeau reports grants from National Institute of Allergy and Infectious Diseases (NIAID), National Heart, Lung, and Blood Institute (NHLBI), National Institute of Environmental Health Sciences (NIEHS), and Food Allergy Research & Education (FARE); Stock options from IgGenix, Seed Health, ClostraBio, Cour, Alladapt; Advisor at Cour Pharma; Consultant for Excellergy, Red tree ventures, Before Brands, Alladapt, Cour, Latitude, Regeneron, and IgGenix; Co‐founder of Before Brands, Alladapt, Latitude, and IgGenix; National Scientific Committee member at Immune Tolerance Network (ITN), and National Institutes of Health (NIH) clinical research centers; patents include, “Mixed allergen composition and methods for using the same,” “Granulocyte‐based methods for detecting and monitoring immune system disorders,” and “Methods and Assays for Detecting and Quantifying Pure Subpopulations of White Blood Cells in Immune System Disorders.” Dr. O'Mahony reports grants from Science Foundation Ireland, during the conduct of the study; grants from GSK, grants from Chiesi, personal fees from PrecisionBiotics, personal fees from Nestle, personal fees from Reckitt, outside the submitted work. Dr. Akdis has received research grants from the Swiss National Science Foundation, European Union (EU CURE, EU Syn‐Air‐G), Novartis Research Institutes, (Basel, Switzerland), Stanford University (Redwood City, Calif), Seed Health (Boston, USA) and SciBase (Stockholm, Sweden); is the Co‐Chair for EAACI Guidelines on Environmental Science in Allergic diseases and Asthma; Chair of the EAACI Epithelial Cell Biology Working Group; is on the Advisory Boards of Sanofi/Regeneron (Bern, Switzerland, New York, USA), Stanford University Sean Parker Asthma Allergy Center (CA, USA), Novartis (Basel, Switzerland), Glaxo Smith Kline (Zurich, Switzerland), Bristol‐Myers Squibb (New York, USA), Seed Health (Boston, USA) and SciBase (Stockholm, Sweden); and is the Editor‐in‐Chief of Allergy. The remaining authors declare no competing interests.

## Supporting information


Data S1

## Data Availability

The data that support the findings of this study are available from the corresponding author upon reasonable request.
