# Supplementary material for: Rural and urban exposures shape early life immune development in South African children with atopic dermatitis and nonallergic children
Source: Allergy. 2023 Aug 3;79(1):65–79. doi: 10.1111/all.15832 (PMC10952395; doi:10.1111/all.15832)

**SUPPLEMENTARY MATERIAL**

**Supplementary Table S1.** Participant Demographics

|  | **Rural AD** | **Rural HC** | **Urban AD** | **Urban HC** |
| --- | --- | --- | --- | --- |
| N= | 46 | 44 | 33 | 27 |
| Age [months] | 21.0  (15.8 – 25.0) | 20.1  (15.0 – 35.0) | 22.5  (16.0 – 28.0) | 24.4  (18.0 – 34.0) |
| Gender (F/M) | 20/26 | 19/25 | 14/17 | 12/15 |
| Height [cm] | 80.0  (76.8 – 85.3) | 84.0  (79.0 – 87.0) | 81.5  (77.5 – 84.8) | 85.0  (78.0 – 89.0) |
| Weight [kg] | 11.7  (10.5 – 13.2) | 12.0  (10.8 – 13.0) | 12.0  (10.3 – 13.1) | 12.5  (10.5 – 14.6) |
| SCORAD | 43.0  (37.4 – 51.6) |  | 43.5  (36.1 – 55.3) |  |

AD – Atopic Dermatitis; HC – Healthy Control

Results are shown as median with the 25% - 75% percentile range in brackets.

**Figure S1.** PCA of participants with atopic dermatitis and their skin prick test reactivity.

**Figure S2.** AHR and AHRR gene expression. Genes are expressed as transcripts per million (TPM) and adjusted p value statistical significance indicated by asterix.


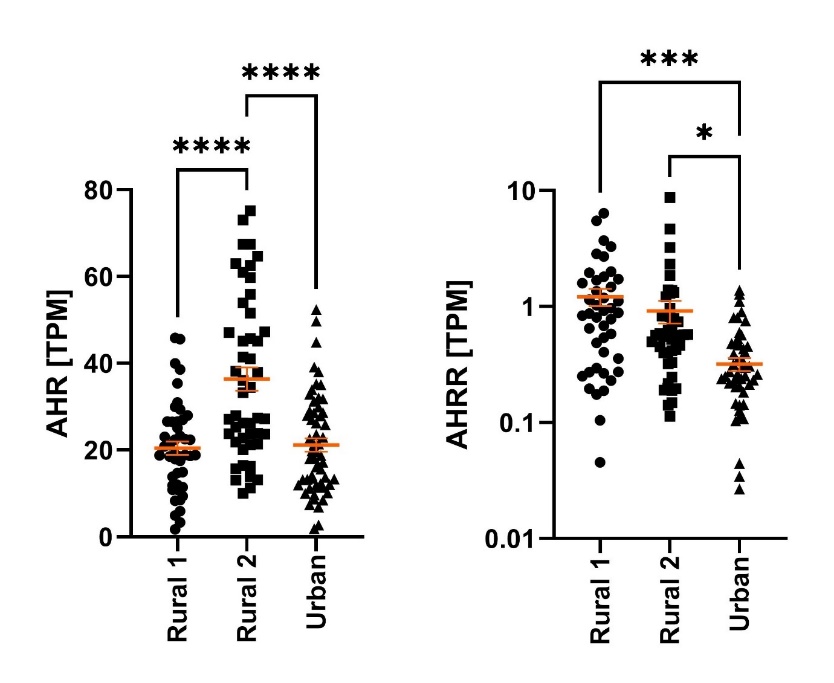

Supplement: Supplementary file 1 — Data S1 [file ALL-79-65-s001.docx]
